# PLGA-Curcumin Attenuates Opioid-Induced Hyperalgesia and Inhibits Spinal CaMKIIα

**DOI:** 10.1371/journal.pone.0146393

**Published:** 2016-01-08

**Authors:** Xiaoyu Hu, Fang Huang, Magdalena Szymusiak, Xuebi Tian, Ying Liu, Zaijie Jim Wang

**Affiliations:** 1 Department of Biopharmaceutical Sciences and Cancer Center, University of Illinois, Chicago, Illinois, United States of America; 2 Department of Chemical Engineering, University of Illinois, Chicago, Illinois, United States of America; 3 Department of Anesthesiology, Tongji Medical College, Huazhong University of Science and Technology, Wuhan, China; University of Kentucky Medical Center, UNITED STATES

## Abstract

Opioid-induced hyperalgesia (OIH) is one of the major problems associated with prolonged use of opioids for the treatment of chronic pain. Effective treatment for OIH is lacking. In this study, we examined the efficacy and preliminary mechanism of curcumin in attenuating OIH. We employed a newly developed PLGA-curcumin nanoformulation (PLGA-curcumin) in order to improve the solubility of curcumin, which has been a major obstacle in properly characterizing curcumin’s mechanism of action and efficacy. We found that curcumin administered intrathecally or orally significantly attenuated hyperalgesia in mice with morphine-induced OIH. Furthermore, we demonstrated that the effects of curcumin on OIH correlated with the suppression of chronic morphine-induced CaMKIIα activation in the superficial laminae of the spinal dorsal horn. These data suggest that PLGA-curcumin may reverse OIH possibly by inhibiting CaMKIIα and its downstream signaling.

## Introduction

Opioids are widely used as analgesics for moderate to severe pain in clinical practice. Prolonged use of opioids is associated with a number of side effects including drug dependence and tolerance. A problem receiving less attention is the development of paradoxical pain that is known as opioid-induced hyperalgesia (OIH) [1]. Although the underlying mechanism of OIH is still unclear, previous studies from our laboratory have shown that Ca^2+^/calmodulin-dependent protein kinase IIα (CaMKIIα), which is highly expressed in the superficial dorsal horn of the spinal cord, plays a key role in OIH [2]. It has been shown that development of mechanical allodynia and thermal hyperalgesia is correlated with a surge in spinal CaMKIIα activation in OIH. Moreover, chemical inhibition, siRNA knock-down, and genetic deletion of CaMKIIα are highly effective in preventing and/or attenuating OIH [2]. A logic question is how would these findings be translated into clinically useful therapies. Since clinically useful CaMKIIα-specific inhibitors are still in early chemical development, we turned our research attention to currently available botanical dietary supplements that may inhibit CaMKIIα.

*Curcumin longa*, commonly known as turmeric, is a perennial herb in the ginger family and is a commonly used seasoning spice and medicinal plant in Asia for thousands of years. Turmeric has been used as a traditional therapy for a range of diseases and conditions, including inflammatory conditions. Curcumin [1,7-bis-(4-hydroxy-3-methoxyphenyl)-1,6-heptadiene-3,5-dione], also known as curcumin I, is the most active constituent in the rhizome of *Curcuma longa*. While a number of actions have been associated with curcumin including antioxidant, anti-inflammatory, chemotherapeutic and neuroprotective actions [3,4], in all cases, high doses or concentrations of curcumin were required. The latter is problematic, because curcumin is not soluble in aqueous media at high concentrations, which significantly hinders the proper interpretation of study findings [5,6]. Poor bioavailability due to poor solubility and absorption negatively impacts its research, clinical use and therapeutic development. To overcome the problem, we have designed and prepared nanoformulated curcumin (PLGA-curcumin) that is highly soluble and stable [7] and orally available [8]. Moreover, recent studies showed that curcumin directly inhibits Ca^2+^-dependent and -independent activities of CaMKII *in vitro* [9,10], and our preliminary data suggest that curcumin may have an inhibitory action on CaMKIIα *in vivo* [8]. In the current study, we investigated the possible role of curcumin on OIH and CaMKIIα in the spinal cord.

## Materials and Methods

### Materials

Morphine sulfate was obtained from Hospira (Lake Forest, IL). Curcumin, PLGA (acid terminated; PLA:PGA 50:50 w/w; Mw 7000–17000), tetrahydrofuran (THF), and all other chemicals were purchased from Sigma-Aldrich (St. Louis, MO).

### Production of PLGA-curcumin

PLGA-encapsulated curcumin was generated by a multi-inlet vortex mixer (MIVM) method as previously described [7]. PLGA-nanoparticle suspension was freeze-dried and stored. Prior to the experiments, PLGA-curcumin was re-suspended homogeneously using bath sonication. Drug loading, encapsulation efficiency of curcumin in nanoparticles, and nanoparticle size and size distributions were measured as described previously [7].

### Animals

Male ICR (Institute of Cancer Research) mice weighing 20–25g (Harlan Laboratories, Indianapolis, IN) were housed in groups of 5 mice per cage in a standard animal facility on a 14h/10h (light/dark) cycle. Mice were provided with food and water *ad libitum* prior to experimental procedures. Mice were handled and habituated to our animal facility for at least 24 h before use to allow for acclimation, and were monitored every day throughout the experiments. Unformulated curcumin was injected intrathecally *(i*.*t*.*)* or by intragastric gavage *(i*.*g*.*)*. PLGA-curcumin was given orally by intragastric gavage. In total 114 male ICR mice were used in this study. Mice were euthanized by CO_2_ from a bottle source at the end of experiment. Experiment protocols were approved by the University of Illinois Institutional Animal Care and Use Committee and were in accordance with the policies and recommendations of the International Association for the Study of Pain (IASP) and National Institutes of Health (NIH) guidelines for handling and use of laboratory animals.

### Opioid-induced hyperalgesia model in mice

OIH was established in mice as described previously [2]. Mice were treated with morphine sulfate (20mg/kg, s.c.) twice a day at 9:00 a.m and 17:00 p.m. for 3 consecutive days and on day 4 mice received a higher dose of morphine sulfate (40mg/kg, s.c.) twice at 9:00 a.m and 17:00 p.m. Control mice received the same numbers of injection with equal volume of saline.

### Assessment of mechanical sensitivity

Mechanical sensitivity was detected using calibrated von Frey filaments (Stoelting, Wood Dale, IL) as previously described [11,12]. In brief, mice were placed on wire mesh platforms in separate Plexiglas containers and allowed to acclimate for 30 min before testing. The mechanical threshold was assessed by perpendicularly applying von Frey filaments with different forces (0.04g to 4g) to the mid-plantar surface for 5s or until a withdrawal response was observed and calculated using the up and down paradigm.

### Assessment of thermal sensitivity

To determine thermal sensitivity, a radiant heat beam was focused on the middle portion of the plantar surface, using a plantar tester (UGO Basile, Stoelting, Wood Dale, IL), until automatically turned off as paw withdrawal occurred. A cutoff time of 20s was applied to prevent tissue damage. The latency of paw withdrawal was recorded after a 30 min period of habituation as the thermal threshold [11,12].

### Immunoblotting

Spinal CaMKIIα activity was assessed by immunoblotting analysis using tissues from lumbar spinal section. As described previously, tissues were processed in ice-cold radioimmunoprecipitation assay (RIPA) buffer [2,13]. After homogenizing and centrifuging, solubilized samples were separated by 12% SDS-polyacrylamide gel electrophoresis, and electrotransferred onto a polyvinylidene difluoride membrane. After incubation with a rabbit anti-Thr286-pCaMKIIα antibody (1:1,000; Santa Cruz Biotechnology, Inc., Santa Cruz, CA) and a horseradish peroxidase-conjugated donkey anti-rabbit secondary antibody (1:10,000; Thermo Fisher Scientific, Waltham, MA) for pCaMKIIα, or a mouse anti-β-actin antibody (1:10,000; Santa Cruz Biotechnology, Inc.) and a horseradish peroxidase-conjugated donkey anti-mouse secondary antibody (1:10,000; Thermo Fisher Scientific) for β-actin, an enhanced chemiluminescence detection system (ECL; Thermo Fisher Scientific, Waltham, MA) was applied for detection. The specificity of the anti-(T286)-pCaMKIIα antibody was validated in the current study using the tissue taken from the transgenic mice with the CaMKIIαT286A point mutation. ECL signals were detected by a ChemiDoc system and analyzed with the Quantity One program (Bio-Rad, Hercules, CA). Densitometry ratio (arbitrary unit) over actin was first calculated, and was further normalized to that of control.

### Immunofluorescence

Immunoreactivity of pCaMKIIα was detected by immunofluorescence as described before. Mice were deeply anesthetized with ketamine (100mg/kg, i.p.) and xylazine (5mg/kg, i.p.), and the vascular system was perfused with 10ml of 4°C PBS (pH 7.4), followed by 20ml of 4% paraformaldehyde solution [2]. The lumbar spinal cord was dissected and post-fixed in 4% paraformaldehyde at 4°C for 18h, and cryoprotected in 20% sucrose for 24h at 4°C. The tissue was sliced to 20μm thickness using a cryostat and rinsed with cold PBS twice for 5min. Floated sections were incubated with a rabbit anti-Thr286-pCaMKIIα antibody (1:1,000; Santa Cruz Biotechnology, Inc., Santa Cruz, CA) and an Alexa Fluor 488-conjugated secondary antibody (Jackson Immunoresearch Inc., West Grove, PA). The fluorescence signals of the spinal cord sections were imaged by microscopy (Olympus, Center Valley, PA) and quantified using the MetaMorph Software (Universal Imaging, Bedford Hills, New York).

### Statistical analysis

All data are presented as mean ± S.E.M. Comparisons between groups were analyzed using a one-way analysis of variance followed by Tukey’s post hoc tests or a two-way analysis of variance followed by Bonferroni post hoc tests. Statistical significance was established at a 95% confidence limit.

## Results

### Intrathecal curcumin ameliorated morphine-induced hyperalgesia

**Fig 1:**
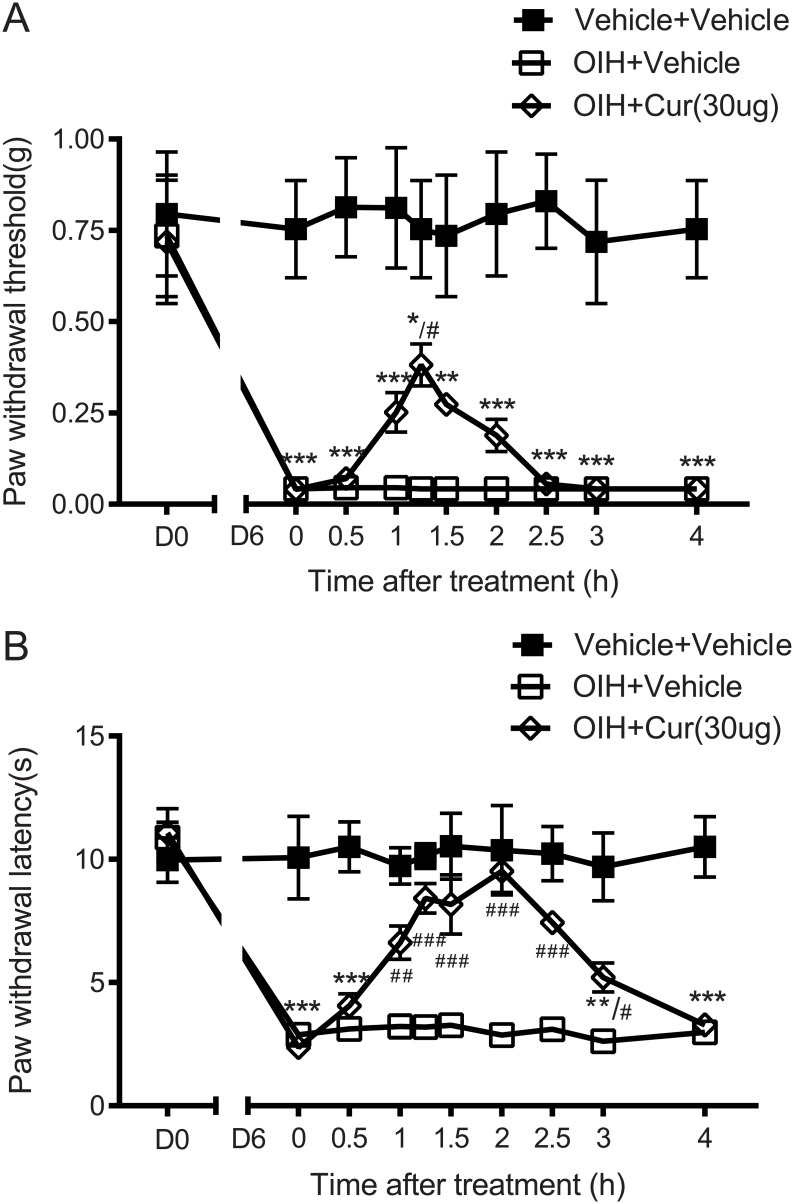
Attenuation of morphine-induced mechanical allodynia (A) and thermal hyperalgesia (B) by intrathecal curcumin. Separate groups of six mice received repeated morphine administration to induce OIH. Mice developed mechanical and thermal hyperalgesia gradually compared with saline treated mice. On day 6, mice were treated with curcumin (30μg, i.t.) or vehicle control. Sensitivities to mechanical and thermal stimuli were determined at the different time points as indicated. Curcumin (30μg, i.t.) partially reversed the established morphine-induced mechanical allodynia, and fully blocked thermal hyperalgesia. Data are expressed in Mean ± SEM. *, p<0.05; **, p<0.01; ***, p<0.001 compared with vehicle+vehicle group; #, p<0.05; ##, p<0.01; ###, p<0.001 compared with the OIH+vehicle group.

OIH was established in mice by repeated subcutaneous morphine administration. Mechanical (0.04±0.01g OIH vs 0.75±0.13g Sham, p<0.001) and thermal (2.88±0.32s OIH vs 10.07±1.68s Sham, p<0.001, Fig 1B) nociception thresholds were significantly decreased in OIH mice compared with saline treated mice. We first delivered curcumin directly into the spinal cord to test whether the central action of curcumin produced an anti-hyperalgesic effect in OIH mice. Acute treatment with curcumin (30μg, *i*.*t*.) partially reversed the mechanical allodynia (0.38±0.06g, p<0.05, Fig 1A), and completely blocked the thermal hyperalgesia (9.52±0.87s, p<0.001, Fig 1B). These data indicated that intrathecal curcumin is effective in attenuating morphine-induced hyperalgesia, especially thermal hyperalgesia.

### Oral administration of PLGA-curcumin reversed opioid-induced hyperalgesia

**Fig 2:**
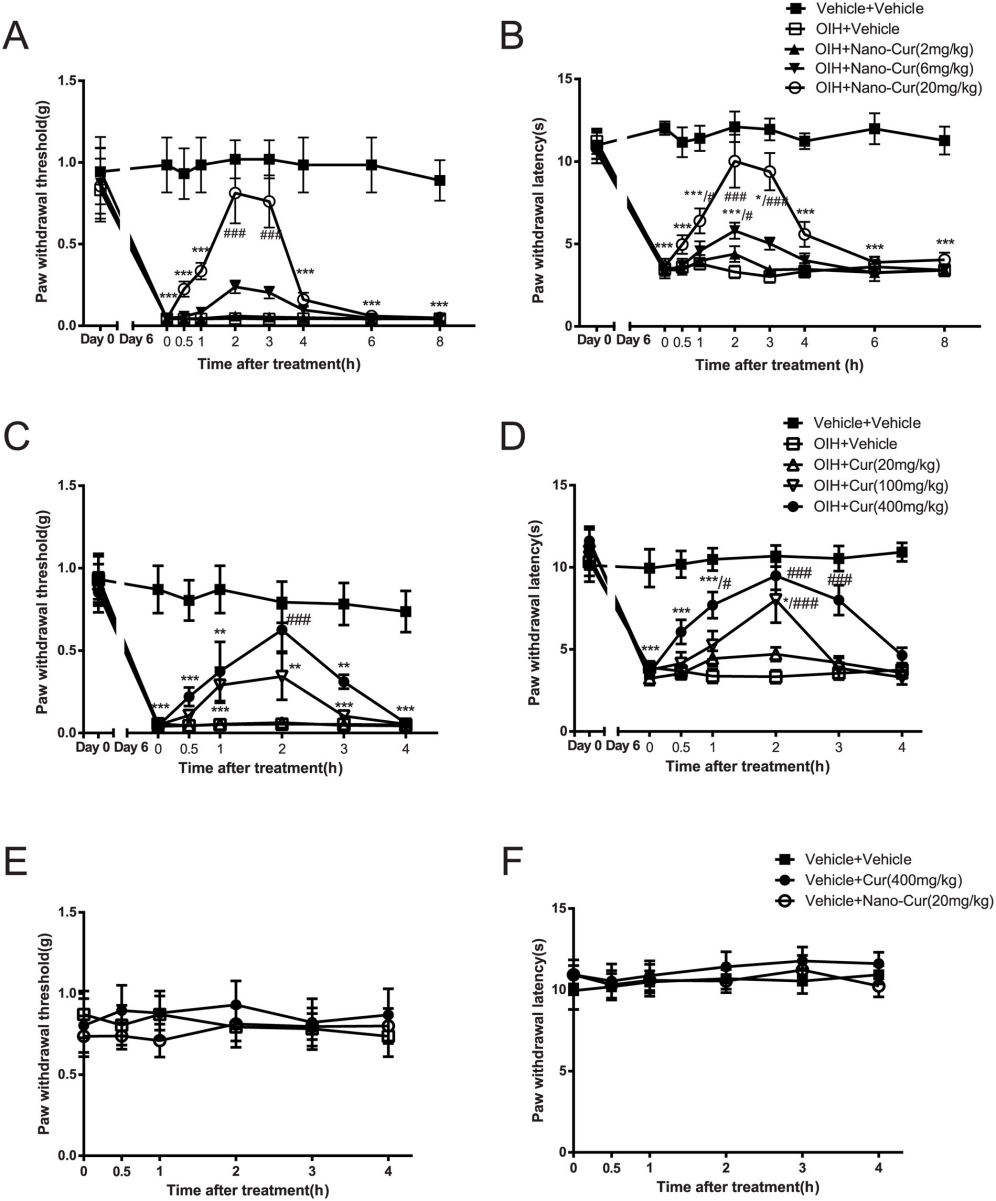
Reversal of morphine-induced mechanical allodynia and thermal hyperalgesia by PLGA-curcumin and non-formulated curcumin. Separate groups of six to eight mice were given subcutaneous intermittent morphine injections to induce OIH. PLGA-Curcumin (2–20mg/kg, i.g.) dose-dependently reversed morphine-induced both (A) mechanical allodynia and (B) thermal hyperalgesia in OIH mice. Non-formulated curcumin (20–400mg/kg, i.g.) attenuated (C) mechanical and (D) thermal hypersensitivities at larger doses. Another groups of six to eight naïve mice received PLGA-curcumin and non-formulated curcumin. PLGA-curcumin (20mg/kg) and non-formulated curcumin (400mg/kg) alone do not change (E) mechanical and (F) thermal sensitivities in mice. Data are expressed in Mean ± SEM. *, p<0.05; **, p<0.01, ***, p<0.001 compared with vehicle+vehicle group; #, p<0.05, ###, p<0.001 compared with the OIH+vehicle group.

Although curcumin was effective in reversing already-established OIH, only a partial effect on mechanical allodynia was obtained at the high dose used. The drug’s solubility issue limited further increase of its concentrations to obtain the maximal effect in order to construct a dose-response curve. We therefore turned to curcumin nanoparticles (PLGA-curcumin) and investigated whether PLGA-curcumin with improved solubility and bioavailability, can be effective in attenuating OIH when given orally. Mice treated with 4-day intermittent morphine (s.c.) developed significant mechanical allodynia (0.04±0.01g OIH vs 0.99±0.17g Sham, p<0.001, Fig 2A) and thermal hyperalgesia (3.48±0.34s OIH vs 12.02±0.41s Sham, p<0.001, Fig 2B), compared with the saline treated group. After OIH was established and morphine treatment had stopped, mice received PLGA-curcumin (2–20mg/kg) or saline by gastric gavage (i.g.) on day 6. PLGA-curcumin (20mg/kg, i.g.) completely reversed both mechanical allodynia (0.81±0.19g PLGA-curcumin vs 0.04±0.01g OIH, p<0.001, Fig 2A), and thermal hyperalgesia (10.02±1.61s PLGA-curcumin vs 3.30±0.38 s OIH, p<0.001, Fig 2B) in OIH. PLGA-curcumin at a lower dose (6mg/kg, i.g.) partially reduced mechanical and thermal sensitivities in OIH mice (0.24± 0.04g, n.s., Fig 2A; 5.80±0.48s, p<0.05, Fig 2B), whereas PLGA-curcumin at the lowest dose (2mg, i.g.) was ineffective (0.06±0.01g, Fig 2A; 4.38±0.48s, Fig 2B).

In order to assess the efficacy of PLGA-curcumin advanced by the nano-formulation, the analgesic effect of non-formulated curcumin was tested in OIH mice to compare with that of PLGA-curcumin. On day 6 of OIH, mice were given non-formulated curcumin (20–400mg/kg, i.g.) or vehicle. However, when mice were treated with non-formulated curcumin at a dose of 20mg/kg, which is the effective dose of PLGA-curcumin, no amelioration of OIH was observed (0.06±0.01g, Fig 2C; 4.71±0.41s, Fig 2D). Mice received the highest dose of non-formulated curcumin (400mg/kg, i.g.) showed significantly attenuated mechanical allodynia and thermal hyperalgesia (Fig 2C and 2D). Curcumin at 100mg/kg was partially effective. These data suggest that PLGA-curcumin is more effective (~10–20x) than non-formulated curcumin in attenuating OIH.

To examine the possible interfering factor that curcumin may alter the basal nociceptive sensitivity in mice, both non-formulated curcumin and PLGA-curcumin were tested in naïve mice. Neither non-formulated curcumin (400mg/kg) nor PLGA-curcumin (20mg/kg) at highest doses altered the baseline sensitivity to innocuous mechanical probing by von Frey filaments (Fig 2E) or noxious heat stimulus by radiant heat (Fig 2F) up to 4 h after administration.

### Duration and reinstatement of analgesic actions of PLGA-curcumin in opioid-induced hyperalgesia

**Fig 3:**
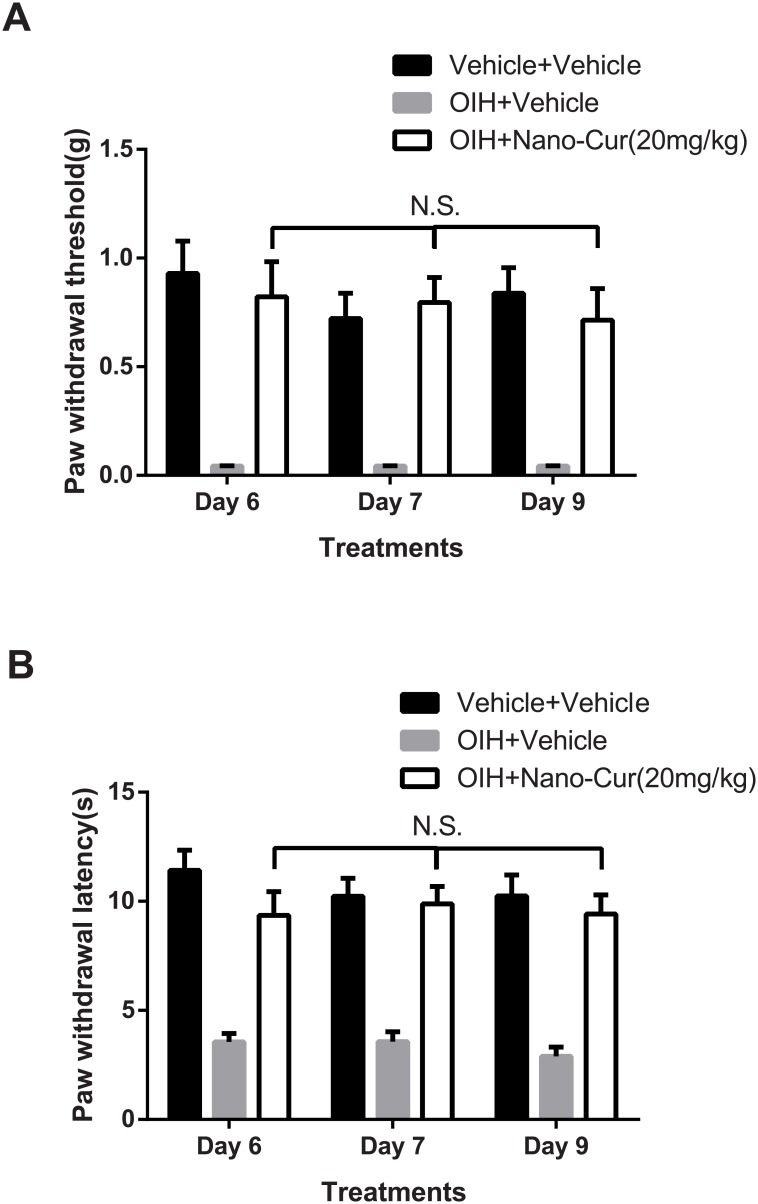
Effects of repeated PLGA-curcumin on OIH. Groups of mice were given repeated morphine to induce OIH. PLGA-curcumin (20mg/kg, i.g.) or vehicle was administrated by gastric gavage on Day 6, 7, and 9 after the initiation of morphine treatment. Data shown were collected at 2h after PLGA-curcumin or vehicle was given. Oral administration of PLGA-curcumin on Day 6, 7, and 9 significantly reversed both (A) mechanical and (B) thermal hypersensitivities in OIH mice. The analgesic effects of PLGA-curcumin on OIH are not significant difference on Day 6, 7 or 9. N.S., p>0.05, compared among groups.

The anti-allodynic/anti-hyperalgesic effects of PLGA-curcumin peaked at 2h and last for at least another hour. By 8 h, hyperalgesia reappeared in mice with OIH. We further tested whether repeated curcumin would reinstate analgesic actions, by administering PLGA-curcumin on day 7 and day 9 of OIH. On either day, PLGA-curcumin was able to reinstate its anti-allodynic/hyperalgesic effects without apparent tolerance (Fig 3).

### PLGA-curcumin reduced the activation of spinal CaMKIIα in mice with opioid-induced hyperalgesia

**Fig 4:**
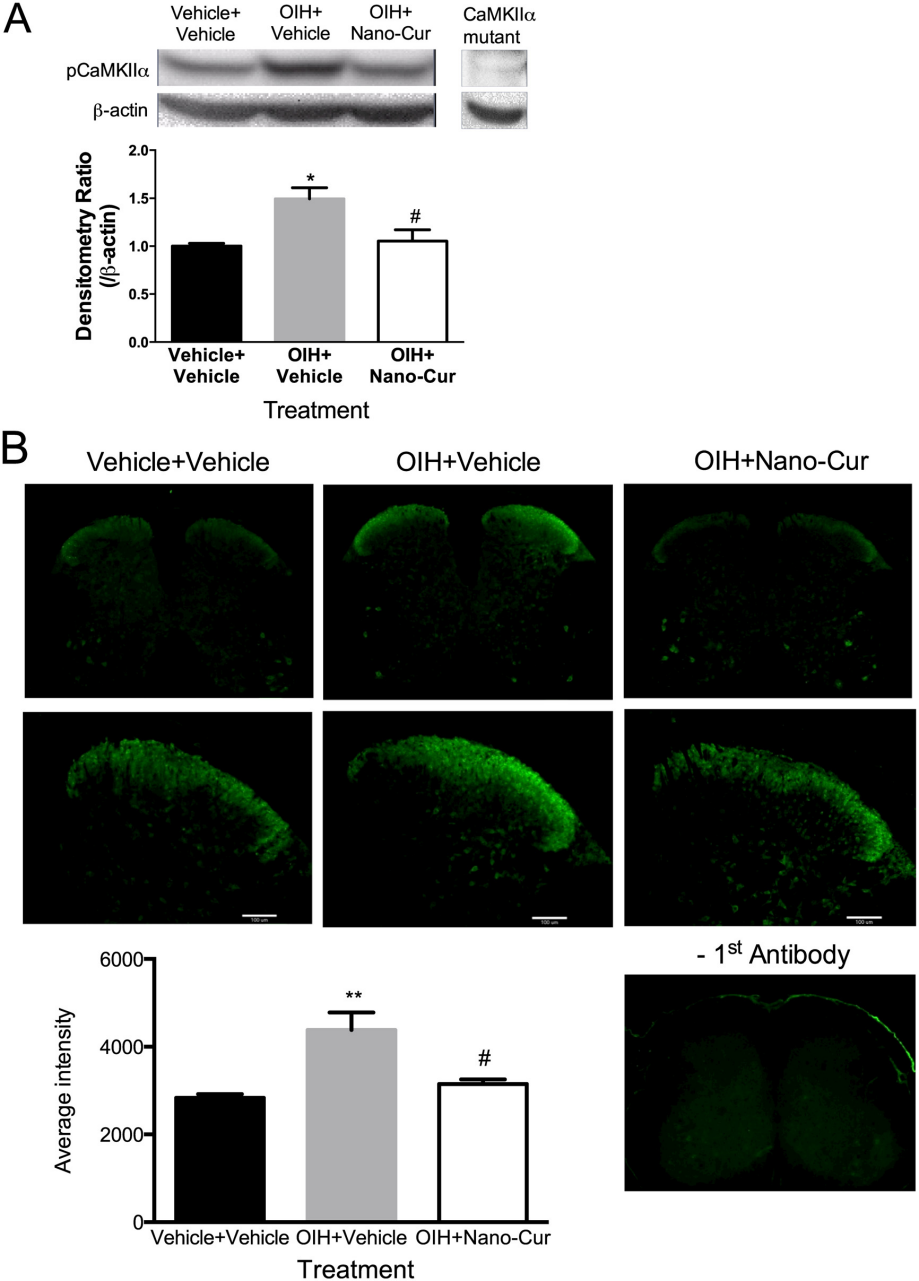
Suppression of morphine-induced CaMKIIα activation by PLGA-Curcumin. Morphine or saline treated mice (n = 3) received curcumin (20mg/kg, i.g.) or saline on day 6. Two hour later, mice were sacrificed and the lumbar spinal cords were carefully removed for the analysis of CaMKIIα activation (pCaMKIIα) using the (A) immunoblotting and (B) immunohistochemistry methods. OIH increased pCaMKIIα, which was reversed by PLGA-Curcumin. Data are expressed in Mean ± SEM. **, p<0.01 compared with the vehicle +vehicle group; #, p<0.05, ##, p<0.01 compared with the OIH+vehicle group.

To investigate the potential molecular mechanism underlying the effect of curcumin in attenuating OIH, CaMKIIα activity in the spinal cord was determined by analyzing the level of its autophosphorylation at T286 (pCaMKIIα), an indicator of its activation, by immunoblotting and immunohistochemistry methods. Chronic morphine exposure significantly increased the activation of CaMKIIα in the spinal cord of OIH mice, compared with the saline control (Fig 4A). Oral administration of PLGA-curcumin (20mg/kg) significantly reduced the level of pCaMKIIα in OIH mice (p<0.05, Fig 4A).

We also examined CaMKIIα activation in the spinal cord using immunohistochemistry. The immunostaining of pCaMKIIα was primarily found in the superficial laminae of the dorsal spinal cord and was significantly enhanced in OIH (Fig 4B). PLGA-curcumin (20mg/kg, i.g.) significantly suppressed pCaMKIIα immunoreactivity in OIH mice (Fig 4B). These data suggest that PLGA-curcumin inhibited spinal CaMKIIα activation, correlating its attenuation of OIH.

## Discussion

In the present study, we found that intrathecal curcumin attenuated the established OIH (especially thermal hyperalgesia) in mice. These data suggested that curcumin can attenuate OIH directly in the central nervous system. The solubility issue of curcumin severely limits performing pharmacological studies with the drug at high concentrations in order to obtain full dose-response spectrum. To better understand the effect of curcumin, we developed PLGA-encapsulated curcumin in nanoparticle form, with significantly improved solubility and bioavailability. Oral administration of PLGA-curcumin (6–20mg/kg, i.g.) significantly attenuated morphine-induced hyperalgesia in a dose-dependent pattern, compared with non-formulated curcumin at the dose of 100–400mg/kg by gastric gavage. Moreover, we correlated the behavioral effects of curcumin on OIH with the inhibition of spinal CaMKIIα activity in mice. The activation of CaMKIIα was significantly increased in mice with prolonged morphine exposure, and OIH-induced CaMKIIα activation was effectively reduced by PLGA-curcumin (20mg/kg, i.g.). These findings are in accordance with our previous studies that have implicated a critical role of CaMKIIα in the development and maintenance of morphine-induced hyperalgesia [2] and opioid tolerance [14–17]. We have shown that CaMKIIα is essential and required for the development and maintenance of OIH, by employing chemical inhibitors of CaMKII, siRNA targeting CaMKIIα and CaMKIIα T286A point mutation mice [2]. Previous studies from our laboratory have also shown that CaMKIIα is key mechanism in the initiation and maintenance of chronic pain [11,18]. Chronic inflammation and spinal nerve ligation-induced persistent pain can be prevented and reversed by inhibiting spinal CaMKIIα [11,18].

Curcumin, a widely used seasoning reagent, has been reported to exhibit anti-oxidant, anti-inflammatory, chemotherapeutic, and anti-nociceptive activities. It has bee found to suppress the nerve injury induced neuropathic pain and diabetic neuropathic pain [19–21], although the underlying mechanism was proposed. Recently, it was reported that chronic treatment with large doses of curcumin prevented the development of OIH in mice, and the effect was associated with the inhibition of histone acetyltransferase (HAT) by curcumin [22]. However curcumin has also been shown to inhibit histone deacetylase (HDAC) *in vitro* and *in vivo* [23–26], an effect that is opposite to the inhibition of histone acetyltransferase.

In this study, we investigated more acute mechanism of curcumin in OIH. In the mice with established OIH, we found PLGA-curcumin (i.g.) or a high dose of curcumin (i.t.) were able to attenuated OIH after a single administration. These data suggest that curcumin can acutely modulate pain pathways through a direct mechanism. It has been recently suggested that curcumin directly blocked CaMKII autophosphorylation in a cell-free system [9], suggesting that curcumin can directly interact and inhibit CaMKII. The direct interaction was also supported by molecular modeling where we found that curcumin is capable of stably binding to the regulatory domain of CaMKII and restraining the binding of calmodulin molecules (S1 Fig).

Besides CaMKIIα, it is likely there will be other mechanisms underlying OIH. In most cases, inhibiting one of these mechanisms can completely attenuate OIH, suggesting that these mechanisms work as a circuitry in vivo to promote or maintain OIH. Several other mechanisms have also been proposed for curcumin, although data are sparse and contradictory in some cases. Curcumin was found to alleviate neuropathic pain by inhibiting the upregulation of brain-derived neurotrophic factor (BDNF) [27]. On the other hand, it has been reported that curcumin protected against glutamate excitotoxicity and produced anti-depressant effect by increasing BDNF levels [28,29]. Curcumin has also been proposed as an inhibitor of nuclear factor-Kappa B (NFκB) and cyclooxygenase-2 (COX-2) [30–32]. However, these proposed mechanisms of action will need to be confirmed by more studies. Having a soluble form of curcumin, such as PLGA-curcumin, will greatly facilitate the mechanistic studies.

In summary, we demonstrated that PLGA-curcumin reversed OIH in mice at relatively low doses, and the effect correlated with the inhibition of CaMKIIα in the superficial laminae of the spinal cord dorsal horn, an area important for pain transmission and processing. This study not only provides a plausible molecular mechanism for the action of curcumin *in vivo*, but also suggests that PLGA-curcumin can be further developed for attenuating opioid-induced hyperalgesia and other chronic pain conditions.

## Supporting Information

S1 Fig Molecular modeling for curcumin and CaMKIIα. The region encompassing the autoinhibitory domain and the Ca2+/Calmodulin binding domain are colored yellow and magenta respectively. Curcumin was shown in orange. Curcumin fits into the regulatory domain of CaMKIIα, restraining the binding of calmodulin molecules. The molecular docking was performed using AutoDock software (version 4.2.6.) [33]. The X-ray crystal structure of CaMKIIα was downloaded from the Protein Data Bank (PDB ID: 3SOA, http://www.rcsb.org/pdb/home/home.do) [34]. The structure of curcumin was obtained from PubChem, NCBI (http://pubchem.ncbi.nlm.nih.gov/) and displayed on PyMOL v1.7. The structures were subjected to energy minimization using AMBER force field.
(PDF)
