# Supplementary figures and images for: PLGA-Curcumin Attenuates Opioid-Induced Hyperalgesia and Inhibits Spinal CaMKIIα
Source: PLoS One. 2016 Jan 8;11(1):e0146393. doi: 10.1371/journal.pone.0146393 (PMC4706327; doi:10.1371/journal.pone.0146393)

S1 Fig. Molecular modeling for curcumin and CaMKII $\alpha$ .

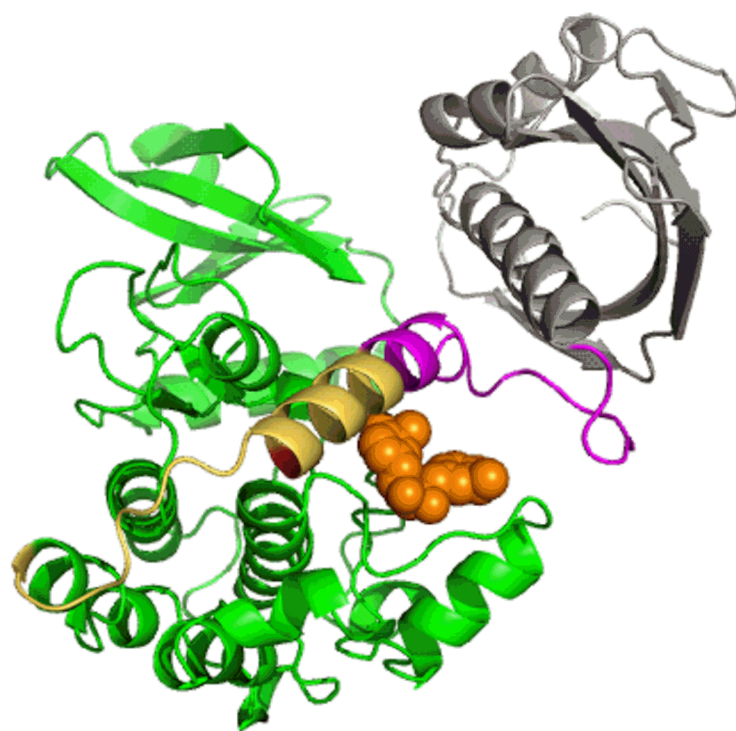

Supplement: S1 Fig — The region encompassing the autoinhibitory domain and the Ca2+/Calmodulin binding domain are colored yellow and magenta respectively. Curcumin was shown in orange. Curcumin fits into the regulatory domain of CaMKIIα, restraining the binding of calmodulin molecules. The molecular docking was performed using AutoDock software (version 4.2.6.) [33]. The X-ray crystal structure of CaMKIIα was downloaded from the Protein Data Bank (PDB ID: 3SOA, http://www.rcsb.org/pdb/home/home.do) [34]. The structure of curcumin was obtained from PubChem, NCBI (http://pubchem.ncbi.nlm.nih.gov/) and displayed on PyMOL v1.7. The structures were subjected to energy minimization using AMBER force field. (PDF) [file pone.0146393.s001.pdf]
